# Rooming-in Reduces Salivary Cortisol Level of Newborn

**DOI:** 10.1155/2018/2845352

**Published:** 2018-03-08

**Authors:** Giuseppe De Bernardo, Marina Riccitelli, Maurizio Giordano, Fabrizio Proietti, Desiree Sordino, Mariangela Longini, Giuseppe Buonocore, Serafina Perrone

**Affiliations:** ^1^Department of Emergency, NICU AORN Santobono-Pausilipon, Naples, Italy; ^2^Department of Molecular and Developmental Medicine, University of Siena, Siena, Italy; ^3^Neonatology and Obstetrics Nursing, C.G. Ruesch, Naples, Italy

## Abstract

**Background:**

Rooming-in practice improves breastfeeding and reduces newborn stress reactivity. When this modality is not available, partial rooming-in after birth can be considered. Salivary cortisol levels (SCLs) are considered reliable biomarkers to indicate stress.

**Objective:**

To test the hypothesis that rooming-in duration impacts neonatal stress response in hospitalized newborns.

**Design/methods:**

Forty term newborns, enrolled in the Neonatology and Obstetrics Nursing, C.G. Ruesch, Naples, Italy, were divided, according to the mother's choice, into the study (SG; *n* = 20) and control (CG; *n* = 20) groups if they received full (24 hs) or partial (14 hs) rooming-in care, respectively. Saliva samples were collected from all babies between 7:00 a.m. and 8:00 a.m. of the 3rd day of life by using oral swab. Salivary cortisol levels were measured using an enzyme immunoassay kit (Salimetrics LLC, PA, USA).

**Results:**

A statistically significant difference in the SCLs between SG and CG was found (median: 258 ng/dl versus 488.5 ng/dl; *p* = 0.048).

**Conclusions:**

Data support the practice of full rooming-in care compared with partial rooming-in. The rooming-in duration clearly reduces SCLs and likely neonatal stress. These lower SCLs may have long-term positive effects reducing the risk of metabolic syndrome, high blood pressure, and cognitive and behavioural changes.

## 1. Introduction

According to clinical managements, newborns are often exposed to painful and stressful situations. The concept of infant pain and effects of pain exposure during hospitalization of infants has progressed greatly in the past 25 years [1]. Grunau et al. and Miller and Ferriero investigated whether neonatal procedural pain/stress impacts the developing brain, discovering that greater exposure to procedural pain-related stress was associated with reduced development of white matter and subcortical gray matter [2, 3]. Therefore, the knowledge of socioemotional stress reactivity during the early period of neonatal life is crucial to understand the brain neurodevelopmental trajectory [4]. When the infant faces up to a stressful situation, the developing hypothalamic-pituitary-adrenal axis is activated: the hypothalamus secretes the corticotropin-releasing hormone, which starts the release of adrenocorticotropic hormone by the pituitary gland. This hormone induces, in the adrenal gland cortex, the secretion of glucocorticoids as cortisol, considered a stress indicator hormone since its plasma levels will be high as a result of the psychological and physical stress response [5]. Long-term side effects of high concentration of cortisol can result in insulin resistance, hyperlipidaemia, immunologic deficiencies, and destructive changes in the hippocampus [6]. Salivary cortisol has been reported as a useful biomarker of psychological stress and related mental or physical diseases [7]. Since Francis et al. in 1987 [8] showed a correlation between infants' plasma and saliva cortisol, only few studies investigated salivary cortisol levels (SCL) due to the complexity of methodology measurement. Morelius et al. published an easy method to collect and analyse cortisol concentration in small amount of saliva [9]. Some studies performed with this method detected patterns of high hormonal stress response to heel lance for infants aged between 34 and 41 weeks of gestation [10]. Infants aged under three months showed increased cortisol levels from baseline in response to painful interventions (e.g., heel lance and inoculation) as well as routine handling interventions (e.g., physical examination, diaper changing, and removal from bath) [11]. Conversely, mother-infant proximity and interactions reduce infants' and mothers' stress during the early postpartum period. Rooming-in practice allows mothers and babies to stay together in the same room during the hospitalization time. Separate care consists in keeping the baby in the hospital nursery and bringing him to the mother only for breastfeeding. These two different approaches may influence mother-infant interaction and neonatal stress response [12].

We tested the hypothesis that full-time rooming-in (for 24 hs) is better than partial rooming-in (for 14 hs) to reduce neonatal stress response in hospitalized newborns.

## 2. Materials and Methods

### 2.1. Enrollment and Group Composition Participants

The study was carried out from January to September 2016, in Neonatology and Obstetrics Nursing, C.G. Ruesch, and Gynaecology and Obstetrics of the Villa Cinzia Hospital in Naples, Italy. Healthy newborns from families that were Italians for more than 3 generations were consecutively recruited while written informed consent was obtained from the families. Eligibility criteria for newborns included patients with gestational age > 37 weeks, 3 days of life, body weight > 2500 gr. Exclusion criteria were patients who underwent painful, stressful procedures, patients born from smoking mothers, mothers with maternal diabetes, placenta abruption, chronic pathologies or using drugs/alcohol during pregnancy, or infants with foetal growth restriction, foetal malformations, chromosomopathy, metabolic disorders, or clinical signs of maternal or foetal infection. Newborns' mothers were <35 years old and primiparous. They had a body mass index between 19 and 24. Infants were divided according to the mother's choice, into the study (SG; *n* = 20) and control (CG; *n* = 20) groups if they received rooming-in care for 24 hs or 14 hs (7:00 a.m.–9:00 p.m.), respectively. The control group (CG) was enrolled from January to June 2016 in Villa Cinzia Hospital and assisted in rooming-in care for 14 hours (7:00 a.m.–9:00 p.m.) as required by the mother. The study group (SG) was enrolled from June to September 2016 in Neonatology and Obstetrics Nursing, C.G. Ruesch, and treated with rooming-in care for 24 hours.

All babies received the same clinical management, the only difference was the rooming-in length. Both hospitals had 20 beds for newborns. Parents could observe clinical bedside round and hold meetings with physicians in the places for rooming-in at 9:00 a.m. in both hospitals. If necessary, families could hold meetings with paediatric nurses every 3 hours. Newborns were not subjected to painful procedures in the first 3 days of life, and during this period, parents could change the diaper, medicate the umbilicus, and breastfeed their children in both hospitals; only the wrapping was performed by paediatric nurses. The environment of the childbirth centres was strictly controlled; the temperature did not have to rise above 25°C, while the luminosity was set at 2000 lux and the noises did not get over 45 dB. The detection of the stress level of the newborns was performed by sampling saliva from 7:00 a.m. to 8:00 a.m. on the 3rd day of life by a paediatric nurse, who was the same throughout the study, to exclude interoperator sampling collecting bias. An oral swab was positioned in the oral cavity for 60–90 sec. to obtain 200–1000 microl. of saliva. The oral swabs were stored in a test tube (stored at a temperature < 20°C) and brought, after two weeks, to the analytical laboratory.

### 2.2. Instruments

During hospital stay of the newborns, one of the authors, aware of the study, recorded clinical features (nationality, parents' residence, gestational age, body weight at birth, days of life in a database (Excel 2007)).

Paediatric nurses who were on duty measured the body weight of the newborns with a seca 354 baby scale. The oral swabs used for this study were SalivaBio Infant's Swab (Salimetrics) while the test tubes were SalivaBio Storage Tube (Salimetrics). The correct collection of the salivary sample considered that patients did not have to eat 60 min before the sampling, the documentation of the oral injury, and vigorous physical activity, as well as, 10 min before the sampling, the cleaning of the oral mucosa with water to remove food leftovers. The cortisol levels in salivary samples were quantified with the Salimetrics Salivary Cortisol Enzyme Immunoassay Kit (Salimetrics LLC, 101 Innovation Boulevard, Suite 302, State College, PA 16803). The method is based on a competitive immunoassay. Cortisol levels were measured in duplicate. Cortisol in standards and samples compete with cortisol conjugated to horseradish peroxidase for the antibody binding sites on a microtiter plate. Bound cortisol enzyme conjugate is measured by the reaction of the horseradish peroxidase enzyme to the substrate tetramethylbenzidine (TMB). The optical density is read on a standard plate reader at 450 nm. In brief, the thawed salivary samples were vortexed and centrifuge at 1500 ×g for 15 minutes to remove mucins ad debris. 25 *μ*l of samples and standard solution with concentration ranging from 0.012 to 3 *μ*g/dl were mixed in a pipette into the plate wells. After, 200 *μ*l of enzyme conjugate cortisol diluted 1 : 1600 in assay diluent was added to all the wells. After an incubation of 1 hour at room temperature, the plate was washed four times with washing buffer. The enzymatic reaction was started by adding 200 *μ*l of TMB Substrate Solution. Mixed on a plate rotator for 5 minutes at 500 rpm and incubated in the dark at room temperature for an additional 25 minutes. The reaction was stopped by adding 50 *μ*l of stop solution and rapidly read at 450 nm with a plate reader. The cortisol levels were determinate by interpolating the optical density on the standard curve obtained with 4-parameter nonlinear regression curve fit. The intra-assay coefficient of variation was 4% at 2.07 *μ*g/dl, and the interassay coefficient of variation was 3% at 1.99 *μ*g/dl. The analytical sensitivity was 0.007 *μ*g/dl.

### 2.3. Statistical Analysis

Analyses of the differences in clinical outcomes were performed by a statistician who was aware of the study aims using IBM SPSS Statistics for Windows, Version 20.0 (Armonk, NY: IBM Corp.). Data with a normal distribution were analysed using the Kolmogorov–Smirnov test. Homogeneity of data groups was assessed using an unpaired *t*-test while the different SCLs between the two groups were analysed by Mann–Whitney *U* test.

## 3. Results

A total of 20 newborns as control group and 20 newborns as study group were recruited. Preliminary data analysis validated the homogeneity of the samples. As showed in Table 1, all newborns and their families were Italian and resided in Campania. There was no difference between the case and study groups for the following parameters: body weight at birth, gestational age, and type of delivery. The sampling of saliva obtained on the 3rd day of life revealed that SCLs were lower in the study group than in the control group (Table 1).

**Figure 1:**
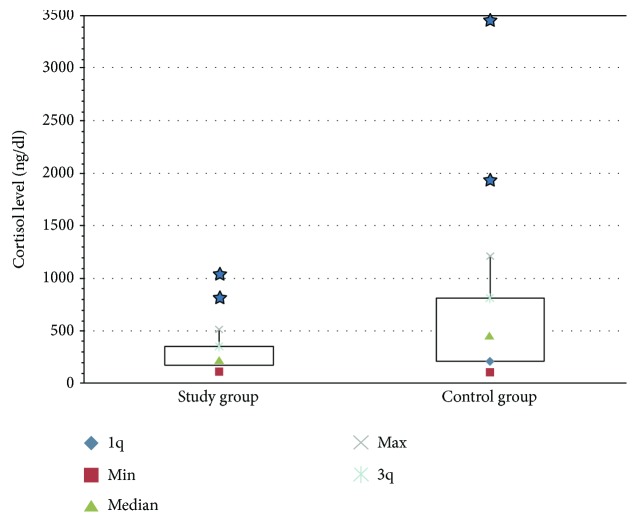
Salivar cortisol levels in the study group and the control group.

There was a statistically significant difference in the SCLs (*p* = 0.048) between the study group (median: 258 ng/dl) and the control group (median: 488.5 ng/dl) (Figure 1).

## 4. Discussion

Keeping the mother and infant together (rooming-in) or separating them after birth are both traditional practices seen in many cultures. Mother-infant proximity during after birth may influence neonatal stress response: this time, shortly after birth represents a sensitive or critical period for programming future physiology and behaviour [13, 14]. According to mammalian neuroscience, rooming-in practice allows the intimate contact that evokes neurobehaviours ensuring fulfilment of basic biological needs [15]. Als suggested that the neurodevelopmental subsystem interaction between the neonate's internal functioning, the environment, and caregivers was the foundation of the neonatal developmental process: if a lack of equilibrium occurs within one subsystem, all other subsystems are affected [16]. Recently, rooming-in has been encouraged also for its benefits both for infants at risk of neonatal abstinence syndrome [17] and for implementation of the duration of breastfeeding [12]. The present paper aims to assess if the presence of the family, using different modalities of rooming-in care, reduces recovery stress by measuring SCLs in hospitalized newborns.

Usually, the procedures practiced are continuous rooming in (for 24 hs), which seems to be preferable, or partial. In literature are no evidence-based reports on which of these two practices is the best. Our work has the purpose to light up this lack of information about these procedures, to minimize pain, and to identify which interventions in hospital reduce stress in infants and to prioritize this intervention in everyday care.

Stress can be defined as a reaction to any real or perceived threat that leads to a disorder in the dynamic equilibrium between the organism and its environment [18, 19]. Newborns are often living and developing in an environment that can be stressful in many ways, starting from the time of birth. An organism's capacity to respond to stressors and maintain homeostasis is regulated by the hypothalamic-pituitary-adrenal (HPA) axis through the cortisol production. Since the beginning of the second trimester, the HPA axis functions and organizes in response to the environment [20]. Different life events, including trauma events, have been shown to influence the HPA, that is, cortisol secretion; therefore, cortisol is considered a major biomarker of stress, also among infants [21]. Recently, it has been showed that prenatal exposure to maternal stress could affect the subsequent cortisol reactivity of the infant [22]. Consequently, we excluded from our study all infants born from a nonphysiological pregnancy or from mothers with chronic pathologies or using drugs/smoke/alcohol. As Taylor et al. affirmed, also the mode of delivery could influence neonatal stress response: they found a lower cortisol reactivity during a procedure among infants delivered by caesarean section compared to those vaginally born [23]. Relying on this, we considered appropriate selecting only newborns born from a caesarean section, to exclude another source of external stress.

Secretion of the cortisol is essential for lung maturation at birth; hence, there is a corresponding increase in cortisol levels with increasing gestational age [21, 24]. Our population consists of full-term newborns, to equalize basal levels of cortisol in all our neonates.

Recently, some authors presented cortisol reference intervals for infants during the first twelve months of life, but only one month after birth, full-term healthy infants develop a circadian rhythm of salivary cortisol with increasing morning levels and corresponding decreasing evening levels [25]. The detection of the stress level in newborns was performed during the morning, in all patients at the same time. There are no cortisol reference values during the first hours of life; thus, it has been possible to establish which of the practice between continuous and partial rooming-in was less stressful only by comparing the cortisol levels between the two groups. Since 1987, cortisol in saliva has been used as a valid method to assess adrenocortical activity in newborns and their response to stressful stimuli [8]: it is an easy and painless method to collect cortisol samples, to analyse neonatal stress. Nowadays, it is possible to collect enough saliva without disturbing the infant, for instance, during sensitive situations, such as mother-infant interaction [6].

In this pilot study, performed in a limited number of newborns, we observed a statistically significant difference between the SG and CG groups in SCLs that strongly suggested that full-time rooming-in is a better practice than partial rooming care in reducing neonatal stress response.

Our data strongly support the practice of rooming-in for 24 hours compared with partial rooming-in. We hypothesize that lower SCLs may have long-term positive effects in reducing the risk of metabolic syndrome, high blood pressure, and cognitive and behavioural changes. The results of this study pave the way for longer RCTs to confirm these preliminary results. Moreover, long-term follow-up studies are needed to verify the long-term effects on brain neurodevelopment.

## Figures and Tables

**Table 1:** Clinical characteristics of study population.

	Control group (*n* = 20)	Study group (*n* = 20)
	Partial rooming-in practice (14 hs)	Full rooming-in practice (24 hs)
Nationality	Italian	Italian
Parents' residence	Campania	Campania
Type of delivery	Elective caesarean section	Elective caesarean section
Maternal age (yrs)	32 ± 2	33 ± 1.5
Maternal BMI	22 ± 2	20 ± 1
Parity	0	0
Gestational age (wks)	38.31 ± 0.63	38.89 ± 1
Birth weight (g)	3072 ± 406	3294.5 ± 357
Days of life at the time of sampling	3	3
Salivary cortisol levels (ng/dl)	727.4 ± 797.18	323 ± 231.8

Data are expressed as mean ± SD. BMI: body mass index.
